# Tourette syndrome research highlights from 2023

**DOI:** 10.12688/f1000research.150931.1

**Published:** 2024-06-24

**Authors:** Andreas Hartmann, Per Andrén, Cyril Atkinson-Clement, Virginie Czernecki, Cécile Delorme, Nanette Mol Debes, Simon Morand-Beaulieu, Kirsten Müller-Vahl, Peristera Paschou, Natalia Szejko, Apostolia Topaloudi, Kevin J. Black

**Affiliations:** 1 Hôpital de la Pitié-Salpêtrière, Assistance Publique - Hopitaux de Paris, Paris, Île-de-France, 75013, France; 2 Department of Psychology, University of Lund, Lund, Sweden; 3 School of Medicine, University of Nottingham, Nottingham, England, UK; 4 Herlev University Hospital, Department of Child Neurology, University of Copenhagen, Copenhagen, Denmark; 5 Department of Psychology, McGill University, Montreal, Québec, Canada; 6 Department of Psychiatry, University of Hannover, Hannover, Germany; 7 College of Science, Purdue University, West Lafayette, Indiana, USA; 8 Department of Neurology, University of Calgary, Calgary, Alberta, Canada; 9 Department of Psychiatry, Washington University in St Louis, St. Louis, Missouri, USA

**Keywords:** Tics; Tourette; annual review

## Abstract

In this, the tenth annual update for the F1000Research Tics collection, we summarize research reports from 2023 on Gilles de la Tourette syndrome and other tic disorders. The authors welcome article suggestions and thoughtful feedback from readers.

## Introduction

This article is the tenth annual update for the F1000Research Tics collection, and is meant to disseminate scientific progress on Tourette Syndrome (TS) that appeared in the year 2023. We searched **PubMed** using the search strategy (“Tic Disorders”[MeSH] OR Tourette) NOT ((Tourette[AU] OR Tourette[COIS]) NOT (“Tic Disorders”[MeSH] OR Tourette [tiab])) AND 2023[PDAT] NOT 1800:2022[PDAT]. On 16 Jan 2024 this search returned 278 citations. A search of **PubMed Central** for “tic disorders”[mesh] OR tourette*[ti] OR tourette*[ab] OR Tourette*[kwd] OR tourettism[tw] AND 2023[dp] NOT 1800:2022[dp] on the same date returned 218 citations, many overlapping. All these citations are available at this link. Colleagues also recommended articles, and we attended selected medical conferences. We selected material for this review subjectively, guided by our judgment of possible future impact on the field. The authors eagerly invite article suggestions for next year’s highlights article at https://www.authorea.com/697735/.

## Main body

### Phenomenology and natural history

**Definition and phenomenology**

Baizabal-Carvallo and colleagues examined 156 patients with TS and 38 patients with secondary tic disorders (of whom some had functional tics) by evaluation of video-recordings and assessment of the clinical history (Baizabal-Carvallo et al. 2023). Compared to patients with secondary tic disorders, patients with TS were more likely to have earlier onset of tics, greater complexity and severity of tics, and tics affecting primarily the head and neck area. Furthermore, patients with TS were more likely to be male. They suggest considering another tic disorder than TS if these characteristics are absent. The same group examined differences between oromandibular tics and tardive dyskinesia. Forty-one patients were identified with oromandibular tics and these were compared with 35 patients with classic tardive dyskinesia. The latter group of patients was found to have more frequent continuous tongue, oromandibular and masticatory movements (Baizabal-Carvallo et al. 2023). According to another study by Baizabal-Carvallo and colleagues, blocking tics occur in 6% of patients (12/201). The most common type of blocking phenomenon was arrest in speech, followed by motor blocking phenomena. There was a correlation between blocking tics and the presence of dystonic tics and the number of phonic tics ( Baizabal-Carvallo and Jankovic 2023).

Several studies have examined gender differences in children and adolescents with tics ( Larsh et al. 2023a, 2024; Nilles et al. 2023a). Gender differences in tic- and non-tic-related impairment were examined in 132 adolescents aged 13-17 years ( Larsh et al. 2023a). Tic- and non-tic-related impairment were higher in adolescent girls then in boys, and in girls parent-reported non-tic-related impairment was correlated with symptoms of OCD. In a second study, sex differences in tic severity by retrospectively reviewing Yale Global Tic Severity Scale (YGTSS) measures in 373 adolescents (13-17 years) before coronavirus disease 2019 (COVID-19) pandemic and during the pandemic ( Larsh et al. 2024). There was no difference in tic severity between the sexes before the pandemic, but during the pandemic where girls had more severe tics than boys. Nilles and colleagues assessed the influence of sex and age on the phenomenology of tics by examining 203 children and adolescents using the YGTSS ( Nilles et al. 2023a). Both were found to affect tics; females had higher frequency and intensity of motor tics than males and this was associated with a greater impairment. Age was positively correlated with total tic severity score.

**Assessment and quantification of tics**

The Motor Tic, Obsession and Compulsion and Vocal Tic Evaluation Survey (MOVES) is an established assessment for tics. Lewin and colleagues evaluated whether only subset of questions can be used as a short screening tool. Both version of the scale demonstrated good sensitivity and specificity was acceptable in comparison to expert assessment ( Lewin et al. 2023).

Two machine learning algorithms for automatic tic detection were evaluated in 64 videos on 35 patients with TS ( Brügge et al. 2023). Tic detection F1 scores (79.5-82.0%) showed that the algorithms are feasible and reliable and might become useful in the assessment and differential diagnosis of tics.

Riechmann and colleagues revised the Rush Video-Based Tic Rating Scale in order to improve the use in research settings ( Riechmann et al. 2023). In total, 102 videos of patients with TS or persistent motor tic disorder were included. Reducing the video time from 10 minutes to 5 minutes did not lead to significant changes in the assessment of tic frequencies. Furthermore, proposed adaptions in anchor values for tic frequency improved correlation with the YGTSS Score, and psychometric properties were acceptable.

**Prognosis and natural history**

In a nationwide cohort study in Sweden, 3761 individuals with tic disorders in childhood were included in order to examine the prevalence and risk factors for the persistence of tic disorders into adult life ( Mataix-Cols et al. 2023). In total, 20% of children with tic disorder received a chronic tic disorder diagnosis in childhood. The strongest risk factors for tic persistence were psychiatric comorbidity in childhood and family history of psychiatric disorders.

**Sensory phenomena and premonitory urge**

In this online study, the authors investigated the nature of non-just-right experiences (NJRE) that have been previously linked to premonitory urges (PU) as well as to obsessive-compulsive disorders (OCD) ( Brandt et al. 2023a). One hundred eleven patients with TS completed different self-assessments for tics, PU and comorbid OCD/obsessive compulsive behaviors (OCB). NJRE were more related to the OCD spectrum than to PU or tics. The same group ( Brandt et al. 2023b) examined clinical characteristics of PU in a large cohort of patients with chronic tic disorders (n=291). PU varied with tic severity. In the vast majority of patients, tics were followed by relief of the urge. ADHD, depression, female gender, and older age were identified as factors associated with the presence of PU. By contrast, OCD and younger age were associated with higher urge intensity.

Larsh and colleagues used a combined approach to determine whether cortical properties such as excitability (CE) as well as cortical inhibition (LICI) correlate with PU and tics. In line with previous studies, PU intensity correlated with tic severity, and higher urge severity correlated with lower CE and LICI ( Larsh et al. 2023b).

Li and colleagues published results of a systematic review and meta-analysis investigating relationship between urge severity and neuronal correlates ( Li et al. 2023). Altogether, 22 studies were identified with a total of 1236 patients. A meta-regression demonstrated that age and tic severity were related to PU severity. From a neuroanatomical perspective, PU was related to the following regions of the brain: insula, prefrontal cortex, anterior cingulate cortex, and supplementary motor area.

A study from Japan explored pre-movement gating (attenuation) using somatosensory evoked potentials (SEPs) ( Kimura et al. 2023). The authors found that sensorimotor processing was preserved for simple tics but impaired for complex tics in a group of individuals after middle adolescence.

**Transient effects of environment on tic severity**

A fascinating study from Israel examined the timing of tics moment to moment while children with tic disorders watched a movie and played a video game ( Raz et al. 2024). Tics did not occur randomly over time but rather were more or less common across participants during specific moments of the movie clip and when reward was expected in the video game. One interesting hypothesis presented for future study was that “the portrayal of motor actions in movies elicits” an urge to tic. Similarly, one would be very interested in whether movie or game conditions eliciting higher tic rates correspond to greater release of striatal dopamine in people with and without tics ( Koepp et al. 1998).

A group from Italy explored the impact of SARS-COV-2 infection in children and adolescents with TS ( Prato et al. 2023). Participants who had COVID-19 infection experienced both short-lasting as well as long-lasting symptoms (“long COVID”). Of note, 35% of patients experienced worsening of tics and/or of psychiatric comorbidities. The impact of the COVID-19 pandemic on tics was also investigated by Hall and colleagues ( Hall et al. 2023). The authors compared YGTSS scores before and during the pandemic in children and young people (N = 112). No significant differences were found between the two assessments.

In a population-based study, Jack and colleagues reported that the incidence of tics in children and young people increased across all age and sex groups during the COVID-19 pandemic, especially in teenage girls ( Jack et al. 2023). However, it is plausible to speculate that many of the patients diagnosed with tics in fact had FTLB, since it is well known that the incidence of FTLB dramatically increased during the pandemic and many of these patients were misdiagnosed with TS.

**Functional tic-like behaviors**

Various authors have identified differences in the frequency or character of premonitory phenomena (PU) as a potential feature that can discriminate functional tic-like behaviors (FTLB) from primary tic disorders ( Malaty et al. 2022; Martino et al. 2023). The frequency of PU in those prior reports differed to a clinically important degree from the frequency in typical tic patients at a similar disease duration ( Arbuckle et al. 2022). However, prospectively comparing 83 patients with typical tics and 40 with FTLB from the Calgary tic registry, Szejko and colleagues found no significant differences in premonitory urge severity (PUTS scale total score) nor in any of the individual PUTS items ( Szejko et al. 2023). The authors noted that their results are supported by other reasonably large case series ( Fremer et al. 2022; Buts et al. 2022), and provide a brief but compelling discussion of potential implications.

Clinical differences between functional tics and neurodevelopmental tics were confirmed in a study by Cavanna and colleagues ( Cavanna et al. 2023a). In this study, 105 consecutive patients who had developed functional tics in the period from April 2020 to March 2023 were examined using a neuropsychiatric assessment. Besides the (sub-) acute onset and high frequency of complex movements and vocalizations, it was shown that 23% of patients had a pre-existing tic disorder, 70% had comorbid anxiety, 40% had a comorbid affective disorder, and 41% had at least one other functional neurological disorder. The same group directly compared the clinical features of patients who developed functional tics during the COVID-19 pandemic (N = 83) to patients with TS matched for age and gender (N = 83) ( Cavanna et al. 2023b). This comparison identified many variables previously reported to differ between the two groups, but the statistically strongest indicators were “tic-related obsessive-compulsive behaviors” and a family history of tics, both of which were much more common in typical TS. Another interesting study on this topic by the same group ( Cavanna et al. 2023c) compared 66 patients with FTLB with 44 patients with other functional movement disorders (FMD), namely functional symptoms suggesting dystonia, tremor, gait disorder, and myoclonus. While both groups shared some characteristics such as female preponderance, comorbid anxiety, depression, other functional neurological symptoms, and subacute onset of symptoms, patients with FTLB had an earlier age of symptom onset and were more frequently exposed to social media than those with other forms of FMD.

Fremer and colleagues ( Fremer et al. 2024) compared a group of patients with FTLB (n=32) with a very large sample of patients with tics (n=1032). A number of previously reported characteristics of FTLB could be replicated: older age of onset, higher proportion of females, and higher rate of obscene and socially inappropriate behaviors. Interestingly, patients with FTLB had significantly lower rates of psychiatric comorbidities typically seen in TS such as ADHD and OCD. Phenotypic differences between patients with FTLB (n=53) and tics (n=200) were also analyzed by a group of researchers from Denmark ( Andersen et al. 2023a). Patients with FTLB were found to have more complex symptoms, were older at symptom onset, were more frequently females, and had less frequently a positive family history for tics. As a new finding, they reported that patients with FTLB had more family members with psychiatric disorders and more often had a history of trauma preceding the onset. In another Danish study, Okkels and colleagues described a cohort of patients with FTLB ( Okkels et al. 2023). Again, most were females, and had mainly complex movements with no rostrocaudal progression. Almost 70% reported harmful behaviors, and 96% had exposure to relevant social media.

As noted above, patients with TS are not immune from developing FTLB in addition to pre-existing developmental tics. This association is not surprising, as for example pseudoseizures are more common in people with epilepsy. Müller-Vahl et al. (2024) presented data on 71 TS patients whom they also diagnosed with FTLB. A majority (56%-79%) had psychological features common in people with other functional symptoms, and about a third of them had a history of other medically unexplained symptoms; these findings suggest that the cause of FTLBs is likely similar to that of other functional neurological symptoms. The authors comment that their ability to identify a fairly large sample of TS+FTLB suggests that clinicians faced with treatment-resistant symptoms in TS should consider whether the symptoms are FTLB rather than tics.

Finally, an international group of experts published results of the so far largest group of patients with FTLB seen in multiple centers across the globe ( Martino et al. 2023). Altogether, 294 patients with FTLB were included. The vast majority were adolescents and young adults (97%), 87% were females, 70% presented with rapid symptom progression, and spontaneous remission was noted in 20% of cases. From the phenomenological perspective, 85% demonstrated complex movements and 81% complex vocalizations. One fifth had a preexisting tic disorder, 66% had anxiety, around 30% depression, 24% autism, and 23% ADHD. Again, a high number (60%) reported exposure to social media.

Diagnostic agreement in assessing FTLB was examined by asking eight experts in diagnosing and treating patients with tics to evaluate videos from 24 adults and diagnose them with either functional tics, primary tics or both ( Rigas et al. 2023). The diagnostic agreement was based on phenomenology alone, and increased to moderate when additional clinical information was provided. However, the diagnostic distinction between primary and functional tics was shown to be difficult in the absence of clinical information.

Regarding long-term prognosis in 83 youth 83 youth with FTLB, Nilles and colleagues observed a meaningful improvement of FTLB over a period of 12 months suggesting an overall good prognosis ( Nilles et al. 2024).

**Comorbidities**

Sadeh and colleagues examined the presence of depressive symptoms in a cohort of 85 children and adolescents with chronic tic disorders (CTD), aged 6-18 years, using the Child Depression Inventory ( Sadeh et al. 2023). In total, 21% had depressive symptoms and the presence of depressive symptoms was correlated with the presence of comorbid OCD and/or ADHD. Furthermore, symptoms of depression moderated the correlation between tic-related impairment and tic severity. Therefore, the authors suggest that it is important to screen and treat depression in children with CTD.

The presence of depression and anxiety were assessed in several other studies. Isaacs and colleagues examined a population of 120 adult patients with CTD with several scales as part of routine care ( Isaacs et al. 2023). Symptoms of anxiety were more common than depressive symptoms. Anxiety, depressive and OCD symptom severity were significantly associated to each other, but not to tic severity. In a systematic review and meta-analysis including twelve studies from 1997-2022, the prevalence of anxiety and depression in TS was estimated at 36.4% and 53.5% respectively ( Abbasi et al. 2023).

Koenn and colleagues examined impulsivity in its multidimensional aspects. They compared 16 patients with OCD, 14 patients with TS and 28 healthy subjects using the self-rated Barratt Impulsiveness Scale BIS-11 (attentional, motor, non-planning) and a continuous performance test assessing sustained attention, working memory, and cognitive impulsivity. Both patients with OCD and patients with TS showed significant deficits in attention via self-assessment, but no difference was observed in the behavioral test. Tic severity was strongly correlated with attentional impulsivity. The authors concluded that a detailed interpretation of the various tools for measuring impulsive behavior is necessary.

Liu and colleagues surveyed behavioral problems in children with tic disorders in order to develop a model for predicting behavioral problems based on sociodemographic and clinical characteristics ( Liu et al. 2023). They used the Achenbach Child Behavior Checklist (CBCL) in a total of 343 children and showed that 30.3% had behavioral problems. The best predictors were age 12 to 16 years, abnormal birth history, an indulgent parenting pattern, parents or close relatives with tics or other psychiatric disorders, and tic severity.

Rizzo and colleagues carried out a systematic review of social cognition studies in several hyperkinetic movements disorders (Huntington’s disease, dystonia, essential tremor and TS) in accordance with PRISMA guidelines ( Rizzo et al. 2023). This meta-analysis of 50 studies revealed impairments in Theory of Mind and social perception in all hyperkinetic movement disorders, as well as impairments in empathy in HD and TS patients. These findings suggest that individuals with TS may exhibit hypersensitivity toward interoceptive experiences associated with social stimuli due to the altered connectivity with striatal-corticothalamic circuits involved in symptom generation.

Colautti and colleagues studied the creative skills of TS patients in order to know if they could help them better manage their symptoms in their daily lives ( Colautti et al. 2023). Creative thinking is generally defined as the ability to generate an idea that is both innovative (or original) and useful (or appropriate). It distinguishes between divergent thinking, which refers to the ability to produce multiple different solutions to open questions, and convergent thinking which involves the search for a single solution to a well-defined problem by appealing to persistence and focus. The study compared a group of 25 patients with TS and 25 healthy matched controls on different experimental creative thinking tasks and questionnaires. The results showed that TS patients outperformed healthy controls in convergent thinking, and that good divergent thinking could enable better coping with tics in everyday life. The authors concluded that creative thinking can serve as a cognitive resource for non-pharmacological interventions.

Sleep problems were explored in two studies. Colreavy and colleagues found that sleep patterns was more impacted by the pandemic in children with TS than in typically developing children ( Colreavy et al. 2023). Using a naturalistic, longitudinal approach, Keenan and colleagues found that children with TS spend significantly more time in bed, have increased sleep onset latency, reduced sleep efficiency, and lower subjective sleep quality compared to healthy controls, whereas sleep time is comparable ( Keenan et al. 2024). In contrast to clinical observations, self-reported tic severity was not related to increased sleep onset latency. More than 80% of children with TS fulfilled diagnostic criteria for a sleep disorder, highlighting the importance for screening for sleep difficulties in clinical routine.

Kurvits and colleagues investigated the prevalence of compulsive sexual behaviors and paraphilic interests in adults with chronic tic disorders ( Kurvits et al. 2023). In contrast to previous reports, these symptoms were found at the same rate as in the general population. There was also no association with the use of antipsychotics, though in other populations case reports have linked partial dopamine agonists to disinhibited behavior. ADHD was a risk factor for paraphilic interests and compulsive sexual behaviors.

The Danish TS Study Group published the results of a longitudinal study investigating substance use in pediatric patients with TS ( Andersen et al. 2023b). Comorbid ADHD and lower socioeconomic status of the guardian predicted higher risk for tobacco smoking, while coexisting OCD was a protective factor.

Tygesen and colleagues compared fine motor skills in children with TS with their healthy siblings and matched healthy controls, but failed to demonstrate any differences between these three groups ( Tygesen et al. 2023).

Nilles and colleagues published a fascinating review on comorbid developmental stuttering (childhood-onset fluency disorder) and TS. Tics and stuttering are the most common ‘habit disorders’. They both have a male predominance and can significantly alter quality of life. Also, they both fluctuate and often go into remission after childhood. Of note, as clinicians seeing individuals with TS know, there are speech-blocking or stuttering-like tics that can be difficult to distinguish from developmental stuttering. More work disentangling the similarities and differences between both disorders is needed ( Nilles et al. 2023b).

Stereotypies are the major hyperkinetic movements disorders associated with tics during the developmental period, and also a major differential diagnosis. Cavanna and colleagues presented a systematic literature review on comorbid tics and stereotypies based on six original studies that were deemed of sufficient quality and size. 23% of patients diagnosed with stereotypies had comorbid tics; conversely, the prevalence of stereotypies in individuals with TS was estimated at 8%. Interestingly, the authors point out the possibility that treatment-refractory repetitive movements in adults with TS could represent persistent stereotypies ( Cavanna et al. 2024).

### Etiology

**Genetics and epigenetics**

In 2023 three studies analyzed rare variants in TS. First, Saia and colleagues performed Array-CGH analysis of 93 phenotypically well-characterized TS cases to explore if the presence of pathogenic copy number variants (CNVs) was related with frequent clinical features. They investigated incidence of dysmorphic features, epilepsy, brain magnetic resonance imaging (MRI) anomalies, intellectual disability, and severity of symptoms in children with TS. They classified the CNVs as potentially causative variants (PC-CNVs) if they were reported to be associated with TS in the OMIM database, and as non-causative CNVs (NC-CNVs) if there was no previous association with TS reported or if they were of unknown significance. They performed statistical analyses to compare children with PC-CNVs, NC-CNVs and without CNVs (W-CNVs), and detected significant differences among the three groups for the occurrence of epilepsy or isolated EEG anomalies, brain MRI anomalies, intellectual disability and IQ ( Saia et al. 2023).

Another study focusing on rare variants was performed by Fincha and colleagues. They performed a co-segregation analysis on 17 multiplex families including 80 TS or tic disorders patients and 44 healthy members. They prioritized 37 rare and possibly pathogenic variants shared by the cases within a family, and three ultra rare variants in two families. These variants are located in multiple genes and the majority of them on introns ( Fichna et al. 2023).

Tsetsos and colleagues performed the largest Tourette Syndrome GWAS meta-analysis to date with a total of 6,133 TS cases and 13,565 controls, including a novel dataset. The increased sample size provided power to detect a novel genome-wide significant locus on chromosome 5q15, upstream of the NR2F1 gene. This locus was also supported by analyses combining eQTL, Hi-C and GWAS data. NR2F1 is a nuclear receptor that is a regulator of transcription. Additional analyses exploring the association of TS polygenic risk score with brain volume data revealed statistically significant associations with right and left thalamus volumes and right putamen volume ( Tsetsos et al. 2023).

Jain and colleagues (2023) used TS GWAS summary statistics from Tsetsos and colleagues ( Tsetsos et al. 2023) to calculate the TS Polygenic Risk Score (PRS) on individuals in the UK Biobank and then performed a Phenome Wide Association Study (PheWAS) to assess the association of TS genetic risk with a wide range of phenotypes (n=2242). They identified significant associations with 57 traits including depression, anxiety disorder, respiratory conditions, type 2 diabetes and heart palpitations. They also performed cross-disorder comparisons of the PheWAS results with OCD, ASD, and ADHD. The study identified shared associations with multiple health and behavioral phenotypes. TS had similar direction of effects for almost all phenotypes with ASD and ADHD, but OCD had an opposite direction of effect compared to TS (for all phenotypes except mental health traits). Then, they performed sex-specific PheWAS for TS, and they found heart palpitations and type 2 diabetes to be significantly associated with TS risk in males but not in females, while diseases of the respiratory system were associated with TS risk in females but not in males.

Wang and colleagues explored the rare maternally-inherited variants on X chromosome in simplex autism families extending their findings also to TS. First, they identified risk-enriched regions (RERs) using microarray data, and then they used whole-exome sequencing data to explore the rare maternally-inherited damaging variants in autism followed by transmission disequilibrium test which pointed to novel autism risk gene, MAGEC3. They applied the same framework to TS and ADHD male probands, and they observed that both traits were enriched for rare damaging variants in RERs, with similar effect sizes to autism. Finally, they estimated that 27.54% of the rare damaging variants carry risk for TS, with similar percentages for the other two traits ( Wang et al. 2023).

Hughes and colleagues used genetic data and measures of psychopathology from Adolescent Brain Cognitive Development (ABCD) and Generation R, as replication, cohorts to explore the relationships of eight psychiatric disorders and cross-disorder Polygenic Scores (PGSs) to dimensional psychopathology in mid-childhood. They observed that the latent neurodevelopmental (NDV) factor PGS, which included loadings from ADHD, autism spectrum disorder, major depressive disorder, and TS, explained more variance across the spectrum of psychopathology than any other disorder-specific or cross-disorder PGS. They also performed gene-based association tests to define the NDV enriched genes (n=68) that were then included in downstream analyses to explore the pathways affecting the risk for childhood psychopathology. The gene ontology (GO) enrichment analysis of these genes did not return any significant results after FDR correction, however tissue expression showed that they were more strongly expressed in the cerebellum followed by cerebral cortical and subcortical regions. Gene expression analysis within the cerebellum from postmortem fetal and postnatal brain tissue, showed that the NDV genes (N = 68) were expressed significantly more strongly prenatally ( Hughes et al. 2023).

Jiang and colleagues ( Jiang et al. 2023) performed a two-sample Mendelian Randomization study to explore the causal relationships between plasma phosphodiesterases and psychiatric disorders, including TS. In the analysis for TS they used GWAS summary statistics from Yu and colleagues ( Yu et al. 2019), and they observed a positive association of PDE5A with TS, while PDE2A was negatively associated with TS, however for both cases the associations were almost at nominal level.

Mahjani and colleagues utilized the Swedish Medical Birth Register to explore the direct additive genetic effect, genetic maternal effect and environmental maternal effect on CTD liability. They identified 6227 individuals with CTD diagnosis and by applying generalized linear mixed models they observed 60.7% direct additive genetic effect, 4.8% genetic maternal effect and 0.5% environmental maternal effect ( Mahjani et al. 2023).

### Pathophysiology

**Neurophysiology**

In 2023, a few studies used EEG to assess brain connectivity patterns in individuals with TS. Jurgiel and colleagues investigated the additive and interactive effects of TS and ADHD on effective connectivity in children ( Jurgiel et al. 2023). They reported additive effects of aberrant effective connectivity in TS and ADHD spanning several frequency bands. Aberrant effective connectivity was mostly found in children with ADHD, who showed reduced effective connectivity across several posterior and occipital-frontal connections. TS was associated with increased connectivity from the left postcentral to the right precuneus and reduced connectivity from the left occipital cortex to the right precuneus. Another EEG study, which was conducted in adults, assessed functional connectivity within and across nodes of the default mode network ( Yang et al. 2023). They found increased beta-band connectivity between the left and right posterior cingulate/retrosplenial cortices, relative to controls. Also, using graph theoretical metrics, they found enhanced gamma-band degree centrality in the left temporal lobe, which was significantly correlated with increased tic severity.

Two EEG studies have investigated proactive control and binding processes in TS ( Wehmeyer et al. 2023; Wendiggensen et al. 2023). In the first study, EEG markers of proactive control and binding were recorded during a cued task switching paradigm in adults with TS ( Wehmeyer et al. 2023). After temporal decomposition, they found an absence of N2 modulation but increased P3 for repeated responses on task switch trials in the C-cluster, which includes intermediate processes between stimulus and response. In the second study, a S1-S2 paradigm was used to assess binding processes in adolescents and adults with TS ( Wendiggensen et al. 2023). Their analyses, which focused on the theta frequency band, revealed that action file binding effects in the control group relied on the superior parietal regions cortex and the precuneus, whereas the superior frontal gyrus was involved in individuals with TS. Of note, both studies support the idea that integration of action in individuals with TS involves different neurophysiological processes, relative to controls.

To test whether altered social behaviors in TS reflected an overactive mirror neuron system, Weiblen and colleagues investigated mu suppression during an empathy for pain task in adults with TS ( Weiblen et al. 2023). Mu suppression is thought to reflect empathic abilities and could serve as a marker of mirror neuron system function. During the experimental task, participants viewed pictures of hand and feet in neutral or painful situations. Study results revealed that adults with TS showed reduced pain-related mu suppression relative to controls, suggesting altered processing of others’ emotional states.

Triggiani and colleagues assessed the sense of volition and the neural antecedents of tics and voluntary movements using a Libet’s clock paradigm ( Triggiani et al. 2023). They asked adults with TS and controls to take note of the time they had the conscious intention to move and the time when they felt the initiation of a voluntary movement. However, groups did not differ on those measures. Those with TS were also asked to judge the timing of the intention and the initiation of tics, but those did not differ from the timing of voluntary movements. While most adults with TS showed a Bereitschaftspotential prior to tics, beta desynchronization was absent in a majority of individuals, suggesting dissociation between both processes. Furthermore, as commented by Gunduz and Ganos, the absence of beta desynchronization prior to tics could possibly help in distinguishing primary tics from functional tic-like behaviors ( Gunduz and Ganos 2023).

Other electrophysiological studies aimed to investigate motor cortical inhibition using transcranial magnetic stimulation. Consistent with previous findings ( Larsh et al. 2023b), Batschelett and colleagues found no difference between children with TS and controls in terms of motor cortex short-interval cortical inhibition ( Batschelett et al. 2023). However, short-interval cortical inhibition was significantly associated with increased tic severity. Schmidgen and colleagues used transcranial magnetic stimulation to evoke the N100 event-related potential, a marker of motor cortical inhibition thought to reflect GABA _B_ receptors functioning ( Schmidgen et al. 2023). They found reduced modulation of N100 by external (different stimulation intensities) and internal (different motor states: movement preparation and execution) modulation in children with TS. These results suggest altered modulation of motor cortical inhibition in TS, which would be related to GABA _B_ processes.

**Neuroimaging studies**

One of the most noteworthy neuroimaging studies of the last year was conducted by Zouki and colleagues ( Zouki et al. 2023). Similar to a study published the preceding year ( Ganos et al. 2022), they combined both lesion-network mapping (derived from cases associated with tic-inducing lesions) and resting-state functional networks obtained in patients with TS. While the precise anatomical localization of the lesions did not reveal a singular site related to tics, the network-based analysis highlighted a neural network implicating the posterior putamen, the caudate nucleus, the globus pallidus externus, and the precuneus. As a second level analysis, this network was found to be functionally disconnected in TS patients with a specific cluster identified in the right frontal white matter and cingulate gyrus. As highlighted in the accompanying scientific commentary ( Ganos and Horn 2023), the robustness of these findings underscores their potential translation into clinical practice, offering promising avenues for the development of targeted therapeutic interventions.

A second notable study, conducted by Kanaan and colleagues ( Kanaan et al. 2023) was of particular interest. Focusing on the role of iron, the researchers compared quantitative susceptibility mapping and serum ferritin levels (both indicators of brain iron content) between 28 individuals with TS and 26 healthy controls. The findings yielded several significant insights: 1) A notable reduction in iron content was observed in TS in the substantia nigra, subthalamic nucleus, striatum, pallidum and dentate nucleus; 2) Interestingly, the severity of tics exhibited a correlation with serum ferritin levels; 3) Further analysis of gene expression patterns revealed associations between iron levels and neurochemical signalling, mitochondrial processes and phosphorylation-related mechanisms. This study is one of the first to highlight the role of iron in TS.

A third research of interest compiled several metrics of functional connectivity based on three different approaches: the classic static functional connectivity, the dynamic one obtained with a sliding window, and independent component analysis (ICA) based connectivity ( Ramkiran et al. 2023). Altogether, and by compiling a large variety of statistical metrics and analyses, the authors underscored the particular significance of metrics obtained with the dynamical approaches, especially related to networks involving the primary motor cortex, the prefrontal-basal ganglia pathway and the amygdala.

Two other studies of interest were conducted on children with TS. One study ( Hsu et al. 2023) investigated microstructural changes, while the other ( Xin et al. 2023) examined alterations in dynamic brain functional networks. The first study ( Hsu et al. 2023) compared two cohorts comprising 30 individuals each (healthy controls versus individuals with pure and treatment-naïve TS). Utilizing diffusion spectrum imaging metrics, with a specific focus on the cortico-striato-thalamocortical network, the authors did not observe any significant alterations in several segments of the network, but they identified that TS was associated with elevated generalized fractional anisotropy in the right frontostriatal tract (which also correlated with tic severity) and in the bilateral thalamic radiation. In the second study ( Xin et al. 2023), temporal properties of functional connectivity were assessed by comparing 36 male individuals with TS to 27 matched healthy controls. Notably, a dysfunctional functional state was identified in TS, correlating with tic severity. This state was characterized by over-connection within the subcortical, sensorimotor, and default mode networks, coupled with under-connection in the salience and executive control networks. Additionally, TS was associated with higher temporal variability in functional networks compared to controls. Although employing different methodologies, both studies converged to underscore the dysfunction of the basal ganglia in TS, thereby enriching our comprehension of brain functioning in children with the disorder.

Finally, using tractography in 58 TS patients and 35 healthy volunteers, Temiz and colleagues showed a significantly increased limbic cortical connectivity in TS patients ( Temiz et al. 2023). In particular, connectivity of the left insular-subthalamic nucleus was positively correlated with higher impulsivity and anxiety scores.

**Animal models**

TS pathophysiology has been linked to a disturbed migration of cholinergic interneurons into the striatum ( Kataoka et al. 2010), disturbed dopaminergic transmission ( Maia and Conceição 2018), and, in rare monogenetic forms, a loss-of-function in the SLITRK1 gene ( Abelson et al. 2005). Du and colleagues attempt to link all three in a mouse model where SLITRK1 si-RNA was injected into the dorsal striatum, and they observed a number of behavioral, neurochemical and electrophysiological abnormalities compatible with TS in humans. They conclude that targeting the function of striatal cholinergic interneurons might represent as a potential therapeutic strategy for TS ( Du et al. 2023).

### Treatment

**Psychological interventions**

Treatment guidelines published by the American Academy of Neurology (AAN) ( Pringsheim et al. 2019) and the European Society for the Study of Tourette Syndrome (ESSTS) ( Müller-Vahl et al. 2022) recommend behavior therapy (BT) as the first-line intervention for TS/CTD *.* Several modalities of BT are currently available, of which Habit Reversal Training (HRT) and its extended form Comprehensive Behavioral Intervention for Tics (CBIT) has the strongest evidence base. Comparatively less support is available for Exposure and Response Prevention (ERP), but this BT modality remains popular among many clinicians and researchers – especially in Europe.

Conelea and colleagues initiated a project involving researchers, clinicians, patients, and families with the aim to identify future priorities for research of BT for TS/CTD ( Conelea et al. 2024). Key research domains were identified through anonymous community surveys. One of these domains concerned the importance of increasing accessibility to BT for patients. Similar to recent years, dissemination of BT has been a recurring theme in TS/CTD research also in 2023. One way of making BT more available is by using a group format, thereby reducing the needed therapist support compared to a regular in-person format. Bekk and colleagues published an open study of 26 participants (20-70 years) using group-delivered CBIT in Norway, the first of its kind in an adult sample ( Bekk et al. 2023). The results showed a large, significant, mean tic severity improvement (YGTSS-TTS) from baseline to a 1-year follow-up ( *d*=1.20). The resources saved in this particular study may however be questioned given that two therapists were present at all times and that each session lasted 180 minutes (about three times longer than the average in-person session as instructed in the manual by Woods and colleagues ( Woods et al. 2008). In an open study conducted in the UK, Hadjii-Michael and colleagues evaluated an intensive group delivery protocol of ERP ( Hadji-Michael et al. 2024). Twenty young participants (8-16 years) with TS or CTD were recruited and received ERP according to the manual by Verdellen and colleagues ( Verdellen et al. 2011), although intensively delivered (3 days + 1 booster day, compared to 12 weekly 1-hour sessions). Results showed a moderate, significant, mean tic severity improvement ( *r*=0.48), suggesting that intensive group-ERP is preliminarily efficacious. Another delivery format with the potential to increase accessibility to BT is videoconferencing. In an open study by Capriotti and colleagues ( Capriotti et al. 2023), 19 youth and 10 adults received CBIT according to the manual by Woods and colleagues ( Woods et al. 2008), albeit via videoconferencing and without booster sessions. Results showed a large, significant, tic severity improvement (YGTSS-TTS) from baseline to post-treatment for youth ( *d*=1.31) and a medium-sized improvement for adults ( *d*=0.66), further adding to the evidence-base for this delivery format. Lastly, internet-delivered BT with therapist-support has been a popular delivery format in recent years. In a long-term follow-up of the ORBIT trial conducted in the UK ( *N*=224) ( Hollis et al. 2023), internet-delivered ERP was shown superior to internet-delivered psychoeducation all through to a follow-up 18 months post-randomization, although with a small effect size at the this last follow-up timepoint ( *d*=0.27). An additional health economic evaluation showed ERP to be cost-effective compared to psychoeducation. Taken together, this study shows that internet-delivered ERP is an efficacious, cost-effective, and durable intervention, although the small effect size may indicate inferiority to in-person BT.

Another domain identified by the survey conducted by Conelea and colleagues was ways to improve treatment outcomes, which in turn may be linked to identifying the underlying working mechanisms of BT ( Conelea et al. 2024). A proposed working mechanism of BT is within-session habituation to aversive sensations preceding tic occurrence (i.e., premonitory urges). In a Dutch study by van de Griendt and colleagues ( van de Griendt et al. 2023), 29 participants with TS (7-59 years) rated premonitory urge intensity at multiple timepoints during 10 in-person ERP sessions. Results showed an increased urge intensity during the first 15 minutes of each session, which then levelled out during the remaining 45 minutes of the session. The authors concluded that the study did not provide support for within-session habituation as a working mechanism for ERP. In a mechanistic study by Morand-Beaulieu and colleagues ( Morand-Beaulieu et al. 2023a), electroencephalography (EEG) was used to collect data on 32 children (8-13 years) participating in an RCT comparing in-person CBIT to a treatment-as-usual condition. Based on another recent study conducted in an experimental setting where the same group identified a brain network in which functional connectivity was increased during tic suppression in children with TS ( Morand-Beaulieu et al. 2023b), the current study aimed at testing whether the same network was involved in treatment response to in-person CBIT. The results showed that functional connectivity during tic suppression at baseline predicted a reduction in vocal tic severity at post-treatment. To conclude, this study provided evidence for a potential overlap between the working mechanisms of tic suppression when used in an experimental setting compared to a clinical setting.

**Pharmacological studies**

To date, no compound has been specifically marketed for the treatment of tics. Ecopipam, a selective D1 receptor antagonist, might be one of the first drugs to be commercialized for this purpose. After two first proof of principle studies ( Gilbert et al. 2014; Gilbert et al. 2018), Gilbert and colleagues have now published the results of a phase 2b trial (D1AMOND study) in individuals with TS ( Gilbert et al. 2023). Seventy six patients were randomized to ecopipam and 77 to placebo. Total tic scores as assessed by the YGTSS were significantly reduced (by around 30%) in the ecopipam group, as well as secondary outcome measures. Importantly, no metabolic side effects were reported. A phase 3 trial is now underway (NCT06021522).

The results of the CANNA-TICS study appeared in early 2023 ( Müller-Vahl et al. 2023). The authors performed a randomized, controlled trial of nabiximols in 97 adults with TS or chronic motor or verbal tic disorder. Befitting the study investigators’ *a priori* view of the literature, people were randomized to drug or placebo in a 2:1 ratio. The primary, predefined efficacy endpoint was a tic reduction of at least 25% on the YGTSS total tic score after 13 weeks of treatment, a magnitude of change recognized as clinically meaningful improvement by an expert panel. The study did not show significant improvement by this measure. However, there were some indications of improvement, including a higher response rate (22% vs 9%) in the nabiximols group, a significantly greater reduction in self-reported tic severity on the Adult Tic Questionnaire, a numerically greater improvement of tics on a standardized video rating scale, and trends for improvement in quality of life and in impairment due to tics. There were no serious safety issues, with side effects of similar severity in 95% of those in the active drug group versus 79% of those in the placebo group (p=.03). Patients with ADHD or with worse general health were most likely to improve. Thirteen percent of patients at the site that enrolled over half the participants reported intentional or accidental unblinding on an end-of-study interview. Of course, other participants likely suspected their drug assignment; a forced-choice blindedness assessment is not reported. In sum, a reasonably large RCT showed hints of superiority for nabiximols over placebo, but the study did not meet the pre-specified treatment target. The authors are to be commended for timely publishing a technically negative study, but one with important clinical implications. In contrast to the findings of the previous study, a small cross-over RCT (N=22) showed significantly more improvement in tics with a THC + CBD combination than with placebo, YGTSS decrease being 8.9 (±7.6) in the active group and 2.5 (±8.5) in the placebo group ( Mosley et al. 2023). The main side effects were cognitive/sedative in nature.

A 2-year naturalistic study with 1,410 participants (1,147 with ADHD, the rest without ADHD) provided important information relevant to the safety of methylphenidate (MPH) in tic patients ( Man et al. 2023). Their main goal was to see if the stimulant, which suppresses appetite, suppressed growth. There was no significant difference in growth among children in or out of the MPH group. Blood pressure and pulse were significantly higher in the MPH group, but only trivially (about 1 mm Hg and 1 beat per minute after correcting for baseline differences). Tic prevalence decreased significantly in all 3 groups, more in the ADHD groups. There was slightly less tic improvement at 12 months in the group taking MPH (< 2 points on the YGTSS) but no significant difference at 6 months. Note that randomized, placebo-controlled studies show that tics actually *improve*, on average, on MPH ( Black 2023). In summary, MPH is relatively safe in ADHD, including in those with tics.

**Neurosurgery**

Several notable papers were published this year about deep brain stimulation (DBS) in TS. Rusheen and colleagues investigated the effect of centromedian (CM) thalamic DBS on modulating dopamine activity in the dorsomedial striatum, using a comprehensive approach combining electrophysiology, electrochemistry, optogenetics and behavioural measurements ( Rusheen et al. 2023). They found that CM DBS evoked synaptic dopamine release and elevated tonic dopamine levels via striatal cholinergic interneurons, while inactivation of D2 receptors reduced clinical response. This pivotal study suggests that tic improvement with thalamic DBS is partially mediated by D2 receptor activation, providing further evidence of the involvement of dopamine dysfunction as a crucial factor for motor tics in TS.

There is ongoing debate around the most relevant target for DBS in TS, the commonly accepted view being that different structures within a common basal ganglia-thalamo-cortical network may serve as potential targets. In a retrospective study using MRI tractography in 21 patients, Avecillas and colleagues investigated the basal ganglia-thalamo-cortical networks associated with tics and obsessive compulsive behaviors (OCB) improvement in patients treated with either anteromedial globus pallidus (amGPi) or thalamic ventral-oralis complex/centromedian (Vo/CM) DBS ( Avecillas-Chasin et al. 2023). The networks associated with clinical improvement of tics consisted of a limbic pallidothalamic network for the amGPi target, and the premotor thalamocortical network for the thalamic target, both of which being part of a larger “limbic-motor interface network”. Notably, analysis of the volume of tissue activated by DBS in non-responders showed that the stimulation missed the tracts associated with this specific network. This study reinforces the idea that stimulating this network either at its origin (amGPi), or terminal fields (Vo) can lead to substantial tic improvement. Improvement in OCB was related to the connectivity between the dorso-medial prefrontal cortex (dmPFC)/dorsal anterior cingulate (dACC) and CM. The fact that tics and OCB improvement may be linked to the stimulation of distinct networks questions the relevance of targeting more than one structure.

Najera and colleagues reported on two patients with severe GTS and OCD who underwent dual-targeting DBS in both the ventral capsule/ventral striatum (VC/VS) for OCD, and posteroventral GPi for tics ( Najera et al. 2023). Both patients experienced sustained improvement in both tics and OCB.

For the first time, a study explored the relationship between the microlesion effect and the anatomic location of implanted DBS leads in the CM nucleus of the thalamus ( Morishita et al. 2023). The microlesion effect refers to the immediate improvement of symptoms after lead implantation and is considered to result from edema, microhemorrhage, and/or the disruption of fibers along the trajectory of the electrodes. All 6 patients with TS included in this study experienced tic improvement related to microlesion effect. Connectivity analyses showed connections between the area related with microlesion effect and the prefrontal cortices and globus pallidus.

The team of Vilela Filho and colleagues has previously suggested that clinical symptoms of TS may be linked to hyperactivity of the globus pallidus externus (GPe), based on animal models and theoretical models of the basal ganglia. In this recent paper, they report for the first time the results of an open clinical trial of GPe stimulation in TS patients ( Vilela-Filho et al. 2023). They found that 10 of the 13 patients responded to surgery (mean global improvement in tics of 64%, mean improvement of OCB 57.8%). They also found significant improvements in depression and anxiety scores. These promising results of GPe DBS remain to be replicated in future studies. Interestingly, they also reported on 2 patients who continued to have excellent tic improvement despite battery depletion, while another patient experienced severe rebound effect. Another case report of a patient with Centromedian/Parafascicular (Cm/Pf) stimulation who experienced severe tic status following battery depletion was published this year ( Parmera et al. 2023). The mechanisms of these ongoing responses or rebound effects remain to be deciphered.

Lee and colleagues published a new study involving 20 patients who underwent DBS of the amGPi ( Lee et al. 2023). Interestingly, they employed quite unusual stiumulation parameters with lower frequency (around 80 Hz), and higher pulse width (around 120 μs) than commonly used. At 1 year, 60% of patients had a >35% reduction on the on the YGTSS. Initial response appeared to predict improvement on the YGTSS at one year, and the effect at 12 months was more pronounced than at 3 months.

**Other treatments**

Increased activity in the left inferior parietal cortex (BA40) appears to be involved in the generation of tics. However, inhibitory repetitive transcranial magnetic stimulation (rTMS) compared to sham and applied to the left prefrontal cortex (BA 40) in 29 adults with TS showed no evidence of benefit on tics ( Paulus et al. 2023).

Two randomized, controlled trials of median nerve stimulation (MNS) for treatment of tics appeared in 2023, following up on the fascinating initial report by Morera Maiquez and colleagues in 2020 ( Morera Maiquez et al. 2020). The first was an in-laboratory crossover trial comparing repeated 1- or 5-minute sessions of 10 or 12 Hz stimulation in 32 people with a CTD ( Iverson et al. 2023). Rhythmic MNS was given on one day for repeated stimulation-on and -off, 1- and 5-minute blocks, and arrhythmic MNS at the same mean frequency on another day. Either produced significant improvement in tics and premonitory urges, to a similar degree. Since only rhythmic stimulation increases contralateral sensorimotor cortex 12 Hz activity on EEG ( Morera Maiquez et al. 2020) or MEG ( Houlgreave et al. 2022), those effects do not reflect the mechanism of the clinical benefit.

The second MNS controlled trial was a home-based parallel-group study of a wristwatch-style stimulation device ( Morera Maiquez et al. 2023). The active treatment (N=45) used unilateral 10 Hz MNS at a current just above the threshold for activating movement of the thumb. A sham condition (N=45) was MNS that began at the same current as the active treatment, but current was smoothly decreased by about half over the next minute. Both treatments were given for a total of 10 minutes (5 blocks of 2 min on, 1 min off) every morning for 4 weeks. Both treatments activated sensory nerve fibers, and participants were adequately blinded. A third, wait-list condition (N=45), was meant to measure the magnitude of the placebo response in the sham treatment group. During MNS, active treatment was superior to sham MNS and to wait-list controls, and interestingly, the latter two groups had approximately equal responses. Surprisingly, even though stimulation was only on for 10 minutes a day, clinical response “offline,” *i.e.,* YGTSS rated over the entire past week, was also clearly more effective with active MNS.

Willford and Deeb published a scoping review on multidisciplinary care in TS. Thirty one articles were considered. Four primary benefits of multidisciplinary care were identified, making it the preferred model advocated by patients, physicians and organizations to improve clinical outcome. However, limitations to this approach and to this approach and lack of empirical evidence were also highlighted ( Willford and Deeb 2023).

**Treatment for tic-like functional symptoms**

Recent years have seen a considerable increase in patients displaying FTLBs, rather than tics as in TS or CTD. Even though researchers have agreed on how these symptoms may be differentiated from tics ( Pringsheim et al. 2023), little is published on how functional tic-like behaviors may be treated. In an Australian case series published by Maxwell and colleagues ( Maxwell et al. 2023), 8 participants (13-20 years) with functional tic-like behaviors received an adapted CBIT intervention. The adaptations included added components from third-wave CBT, with the aim to target both the tic-like symptoms and potential underlying (and triggering) stress. Overall, cases showed large tic severity reductions (YGTSS-TTS) from baseline to post-treatment, indicating preliminary support for the intervention. Larger and controlled studies are warranted.

### Tics, family and society

Pring and colleagues reviewed the topic of stigma in TS ( Pring et al. 2023). Evidence from 47 publications showed lower self-esteem in youth TS/CTD, who experienced more frequent bullying and other kinds of peer abuse. The community environment contributed, including school and work settings and the general public.

Knowledge of TS among teachers was assessed in a study by Sapozhnikov and colleagues ( Sapozhnikov et al. 2023). Differences in knowledge on TS, self-perceived understanding and use of sources of information were examined in 144 teachers of children with TS and 78 teachers of control subjects, using the pilot questionnaire Teacher Understanding of Tourette Syndrome Survey (TUTS). Teachers of children with TS had higher self-perceived understanding, more knowledge, and used more sources of information compared to the teachers in the control group.

Stacy and colleagues gathered information on perceptions about TS among physicians and caregivers and compared the attitudes between different specialists (neurologists, psychiatrists) and caregivers. While physicians consider pharmacotherapy even when tics are slightly bothersome, caregivers have a preference for behavioral interventions ( Stacy et al. 2023).

Fletcher and colleagues evaluated the professional development needs of health care professionals regarding TS ( Fletcher et al. 2023). As a result, healthcare workers reported urgent need to have access to evidence-based webinars and materials about TS and OCD. While 80% of participants had patients with TS and/or OCD in their practice, only 50% had any formal training in this area. As part of the TS OCD Alberta Network, a program consisting of twelve online webinars was delivered.

Diagnostic accuracy in determining tic diagnosis was explored by a group from South Korea ( Sung and Hong 2023). They found that in the vast majority of cases (96.5%), diagnoses of different tic disorders were made correctly.

The International Parkinson’s Disease and Movement Disorders Society Tic and Tourette Syndrome Study Group formed a subcommittee to discuss further barriers to practice guideline implementation among clinicians treating patients with tics ( Martindale et al. 2023). The following barriers were identified: accessibility to specialized care, financial costs or coverage of treatment, neuropsychiatric comorbidities, treatment side effects, and stigma of the disease.

Marino and colleagues explored patients’ experience of accessing support from primary care physicians in the UK ( Marino et al. 2023). Altogether, more negative than positive experiences were reported. Main areas of frustration included lack of exploration of tics or no psychoeducation. Around 20% of participants had problems to get a referral to a specialist. When it comes to specialized care, adults were mainly seen by neurologists, while children mainly were treated by psychiatrists or pediatricians. Only 28% of patients were seen by physicians specialized in tic disorders. The average waiting time for children was 3-6 months but it was much longer for adults. One fifths of people was discharged from the clinic without further support, while 30% reported support by private healthcare.

In “‘No ill will’: Ticcing on Moral Grounds” Curtis-Wendlandt, argues that tics are less blameworthy than other intentional actions, but are often misconstrued as morally salient behaviours ( Curtis-Wendlandt 2023). Bervoets and colleagues published an interesting viewpoint following similar lines of thought and inviting more inclusive patient-based perspective ( Bervoets et al. 2023).

Coleman and Melia investigated the topic of self-identity in females with TS. The methodology was a focused semi-structured interview conducted via Zoom ( Coleman and Melia 2023). Five themes were established: “I’m not normal”, “I just want to be me”, I’m a “people pleaser”, seeing oneself as an “outsider”, and “it’s just part of me … it’s not going anywhere”. Difficulties with self-acceptance and the autonomy to be one’s true self were noted and appeared to be intensified by stereotypical gender roles and attempts to conceal tics. Findings also suggested that personal growth and feelings of mastery can be achieved through embracing TS as part of one’s identity, or recognizing it as just one aspect of the self. Psychological support focused on accepting and living with tics rather than reducing them may benefit this population and is currently difficult to access. Consideration should also be given to improving the availability of support groups where women with TS can meet others like themselves.

An interesting topic of moral decision making in patients with TS was explored by Vicario and colleagues ( Vicario et al. 2023). The authors found higher inclination for utilitarian solutions of moral dilemmas in patients with TS. Of note, TS individuals had more tendency to quantify something as morally wrong or right. The authors concluded that there might be neurobiological correlates of inappropriate behaviors in people with TS that could be an underlying cause of the higher utilitarian moral decision-making.

Wellen and colleagues investigated health care experiences among a sample of caregivers of children with tic disorders using a survey ( Wellen et al. 2023). They found that the majority (70%) of families first consulted their pediatrician/primary care provider, and caregivers reported receiving care in line with current best practice guidelines. However, caregivers in the current sample also perceived a lack of knowledgeability on the part of their first providers.

The topic of stigma and TS was raised by Shiu and colleagues ( Shiu et al. 2023). The authors identified three types of enacted stigmas: traumatic events, confrontations, and subtle mistreatments. While traumatic events were associated with tic severity, subtle mistreatments contributed to impairment of quality of life.

Lund and colleagues investigated the impact of TS on education ( Lund et al. 2023). This was a longitudinal study to assess the educational achievements at different timepoints. Overall, children with TS had a lower passing rate at lower secondary school and high school compared to healthy controls. These disparities were more likely driven by the severity of comorbidities than tic severity.

## Conclusions

### The literature is growing rapidly

**Figure 1.**
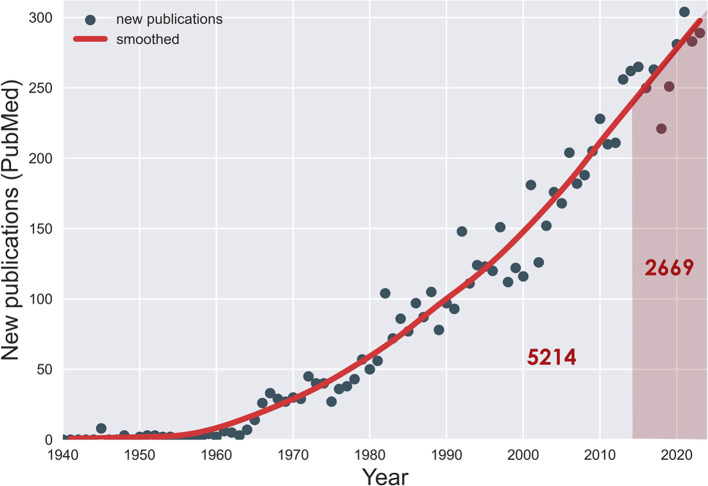
Publications on Tourette syndrome, by year (PubMed). One third of these (2 669) have appeared in the past 10 years.

This is the tenth yearly article in the Tourette Syndrome Research Highlights series. We began this project to highlight top research reports from the past year relevant to TS. Remarkably, one third of all the literature on TS has appeared in just these ten years (see Figure 1). The pace of new research makes it increasingly hard to keep up, and our annual highlight article is getting more voluminous by the year.

New reports this year described important advances in several areas. Increasingly, data have appeared to clarify the phenomenon of functional tic-like symptoms: exactly how they differ from typical tics in TS, their prognosis, and their treatment. Methods for automated detection and timing of tics are progressing steadily and when ripe are likely substantially to aid both research and treatment. Prospective studies of tic outcome continue to be important. The relationship of tics with an urge to tic and sensory phenomena continues to be clarified, along with their electrophysiological substrates. Genetic studies are gradually accruing the large sample sizes required for identifying tic-related genes with small effect sizes. Several EEG studies applied new approaches to study tics.

### Highlights within the highlights

Highlights within the highlights include new treatments for tics that have not yet made it into the clinic but offer hope they will. One includes median nerve stimulation using a portable device (wristband) which would also be the first on-demand treatment of tics ( Morera Maiquez et al. 2023). On the pharmacological aisle, we see the dopamine D1 antagonist ecopipam emerging as the first drug developed specifically for the treatment of tics, with a favorable side effect profile compared to D2 receptor antagonists when it comes to metabolic side effects ( Gilbert et al. 2023). Then, we see studies refining the neuroanatomy and connectivity of TS based on sophisticated neuroimaging approaches and deep brain stimulation treatments ( Zouki et al. 2023). Finally, there is still a substantial literature on FTLB with interesting relations to classic, neurodevelopmental tics that can frequently co-occur, complicating diagnosis and treatment to a certain extent ( Müller-Vahl et al. 2024).

### Scrying the future with a crystal ball

The field seems ripe for additional work in several areas. A number of important questions about tic disorders remain mysterious, and future work may clarify them: Why do tics start around age 5-10 years? Why are boys more likely to develop tics? How accurately can we predict outcomes for individual patients? Why do tics improve during sleep, or with the transition to adolescence and adulthood? Can we identify a natural non-human animal model? Do neurosteroids reduce tics in humans? Which patients need which treatments?

Several areas cry out for new research. The nosology of tic disorders has been driven by historical separations such as whether tics involve muscles of respiration and phonation, or whether tics have been present for 12 months. One can hope for development and acceptance of a more science-based nosology. Acceptance and Commitment Therapy, or other newer behavior therapies that avoid focusing on tic suppression, may be better accepted by patients. Tic disorder therapies have thus far been primarily focused on symptom reduction, but with safe behavior therapies, it is time to consider tic prevention.

Where will the answers come from? Increasing large-scale collaborative studies, including prospective tic surveillance studies, may help bring answers to the clinical mysteries, genetic factors, and treatment development. Novel techniques, such as *ex vivo* organoids from induced pluripotent stem cells, may clarify pathophysiology. We are enthusiastic to see what the next decade brings.

## Data Availability

No data are associated with this article.
