# Supplementary material for: Tourette syndrome research highlights from 2023
Source: F1000Res. 2024 Aug 9;13:677. Originally published 2024 Jun 24. [Version 2] doi: 10.12688/f1000research.150931.2 (PMC11409664; doi:10.12688/f1000research.150931.2)
Supplement: Peer review history

# Reviewer report: Reviewer response for version 2

**DOI:** 10.5256/f1000research.170048.r319056

**Published:** 2024-09-18

**Authors:** Kara A Johnson

**Affiliations:** 1 University of Florida, Gainesville, USA

This is a well written, organized, and approachable review of the latest updates in Tourette syndrome (TS) research focused on several clinical, pathological, and treatment topics. The authors have done an excellent job compiling key research that will be impactful for the field. I enjoy reading this review each year and appreciate the opportunity to participate as a reviewer. I only have a couple of minor suggestions.

 One minor suggestion is more on the stylistic/organizational side. At times the review reads as a list of summaries of the studies and I think it could be enriched by adding a sentence at the end of each paragraph that provides a brief description of the impact of the results on the understanding or treatment of TS. For example, the paragraph about Brandt et al 2023a and 2023b focused on NJRE, PU, and OCD -- it could be helpful to end the paragraph by briefly discussing how these findings could help with stratifying clinical subtypes and provide insight into these behaviors relative to TS.

 Along these lines, I appreciate the "highlights within the highlights" section. Another minor suggestion is I wonder if each section could also have a paragraph that synthesizes the main findings to enable interpretation (almost like a mini-highlights within the highlights)? For example, the FTLB section is excellent but encompasses many studies and it would be helpful to have a few sentences to summarize the key points.

Is the review written in accessible language?

Yes

Are all factual statements correct and adequately supported by citations?

Yes

Are the conclusions drawn appropriate in the context of the current research literature?

Yes

Is the topic of the review discussed comprehensively in the context of the current literature?

Yes

Reviewer Expertise:

Tourette syndrome, neuroimaging, neurophysiology, brain stimulation

I confirm that I have read this submission and believe that I have an appropriate level of expertise to confirm that it is of an acceptable scientific standard.

# Reviewer report: Reviewer response for version 2

**DOI:** 10.5256/f1000research.170048.r312739

**Published:** 2024-09-13

**Authors:** Lorena Fernández de la Cruz

**Affiliations:** 1 Centre for Psychiatry Research, Department of Clinical Neuroscience, Karolinska Institutet, Stockholm, Sweden

I thank the authors for their responsiveness to the questions and suggestions from both reviewers. I believe the minor changes have contributed to improve the article further and I am happy to approve it. Thanks again for inviting me to review this year's TS research highlights!

Is the review written in accessible language?

Yes

Are all factual statements correct and adequately supported by citations?

Yes

Are the conclusions drawn appropriate in the context of the current research literature?

Yes

Is the topic of the review discussed comprehensively in the context of the current literature?

Yes

Reviewer Expertise:

obsessive-compulsive and related disorders, tic disorders, cognitive behavior therapy

I confirm that I have read this submission and believe that I have an appropriate level of expertise to confirm that it is of an acceptable scientific standard.

# Reviewer report: Reviewer response for version 2

**DOI:** 10.5256/f1000research.170048.r312740

**Published:** 2024-08-10

**Authors:** Roger L Albin

**Affiliations:** 1 University of Michigan, Ann Arbor, USA

I thank Dr. Hartmann and his colleagues both for their responsiveness to my comments and the collegial tone of the response. I enjoyed this (indirect) exchange.  RLA

Is the review written in accessible language?

Yes

Are all factual statements correct and adequately supported by citations?

Partly

Are the conclusions drawn appropriate in the context of the current research literature?

Partly

Is the topic of the review discussed comprehensively in the context of the current literature?

Yes

Reviewer Expertise:

Movement Disorders

I confirm that I have read this submission and believe that I have an appropriate level of expertise to confirm that it is of an acceptable scientific standard.

# Reviewer report: Reviewer response for version 1

**DOI:** 10.5256/f1000research.165547.r298068

**Published:** 2024-07-22

**Authors:** Lorena Fernández de la Cruz

**Affiliations:** 1 Centre for Psychiatry Research, Department of Clinical Neuroscience, Karolinska Institutet, Stockholm, Sweden

Thank you for the opportunity to review another informative instance (the 10 ^th^!) of the yearly TS research highlights. I commend the authors for their commitment and for the work itself, which provides a helpful update to both those in the field of TS and curious minds outside of it.

 This is a narrative review restricted to one database (PubMed/MEDLINE), which makes it non-exhaustive but thorough enough for the purpose of the piece. With this in mind, the review follows appropriate methods and critically describes the most relevant published papers in the field of TS/tic disorders, organized by area of knowledge. I do not have major comments but would like to mention some minor points.

 When describing the work by Baizabal-Carvallo and Jankovik (2023), I believe that the reader would benefit from a brief explanation of what the authors mean by “blocking phenomenon”. This is defined in the abstract of the original study (i.e., blocking phenomena are characterized by arrests in motor activity causing interruptions in movements or speech).

 The summary of the work by Mataix-Cols et al (2024) includes a typo which is important for the interpretation of the results. The sentence “20% of children with tic disorder received a chronic tic disorder diagnosis in childhood” should instead read “20% of children with tic disorder received a chronic tic disorder diagnosis in adulthood.”

 When discussing the study by Martino et al (2023) on a large cohort of individuals with functional tic-like behaviors (FTLB), the authors say: “Again, a high number (60%) reported exposure to social media.”  Please clarify that this refers specifically to exposure to tic-related social media content, as mentioned in the original article.

 While reading the piece, I wondered if the focus on FTLB (with one section under “Phenomenology and natural history” and a section under “Treatment”) was not excessive, particularly considering that the authors mention that this review article is becoming more voluminous by the year. The studies on the differential diagnosis between tic disorders and FTLB and their comorbidity are in my opinion very fitting and of help from the clinical point of view. However, other articles focused on samples of FTLB alone, as interesting as they are, may be out of scope (maybe with some exceptions).

 Some acronyms are used but never defined (e.g., ADHD, PUTS, HD). These are most likely well known by the readership but please define for sense of completion the first time that they are mentioned.

 There are several typos throughout the article. It would be good if the authors could read through and correct them. A few that I caught are listed below:

- Description of Larsh et al, 2023a: please write “higher in adolescent girls than in boys” instead of “then in boys”.
- Larsh et al, 2024: “sex differences in tic severity by retrospectively reviewing Yale Global Tic Severity Scale (YGTSS)”. Something seems to be missing in this clause. Maybe rephrase to “sex differences in tic severity were explored by retrospectively reviewing Yale Global Tic Severity Scale (YGTSS)”?
- Larsh et al, 2024: “but during the pandemic where girls had more severe tics than boys”. Remove the “where”.
- Lewin et al, 2023: “only a subset of questions” instead of “only subset of questions” / “both versions of the scale” instead of “both version of the scale”.
- The acronym FTLB is first used when describing the study by Jack et al. (2023) but it is not defined until the following paragraph/section.
- Nilles et al., 2023: “83 youth” is mentioned twice.
- Colreavy et al, 2023: “sleep patterns were more impacted” instead of “sleep patterns was more impacted”.

In sum, I think the article represents a good addition to the TS literature and I recommend its indexing.

Is the review written in accessible language?

Yes

Are all factual statements correct and adequately supported by citations?

Yes

Are the conclusions drawn appropriate in the context of the current research literature?

Yes

Is the topic of the review discussed comprehensively in the context of the current literature?

Yes

Reviewer Expertise:

obsessive-compulsive and related disorders, tic disorders, cognitive behavior therapy

I confirm that I have read this submission and believe that I have an appropriate level of expertise to confirm that it is of an acceptable scientific standard, however I have significant reservations, as outlined above.

# Response

**Published:** 2024-08-05

**Authors:** Andreas Hartmann

**Affiliations:** Pitié-Salpêtrière Hospital, Paris, France

**Response:** We thank Dr Fernández de la Cruz for her thorough review of our yearly review, again ! It is very much appreciated. Below the answers to her queries.

 When describing the work by Baizabal-Carvallo and Jankovik (2023), I believe that the reader would benefit from a brief explanation of what the authors mean by “blocking phenomenon”. This is defined in the abstract of the original study (i.e., blocking phenomena are characterized by arrests in motor activity causing interruptions in movements or speech).

**Response: **Thank you for this suggestion, we have modified the manuscript accordingly.

 The summary of the work by Mataix-Cols et al (2024) includes a typo which is important for the interpretation of the results. The sentence “20% of children with tic disorder received a chronic tic disorder diagnosis in childhood” should instead read “20% of children with tic disorder received a chronic tic disorder diagnosis in adulthood.”

**Response:** Thank you for your vigilance, we have modified the manuscript accordingly.

 When discussing the study by Martino et al (2023) on a large cohort of individuals with functional tic-like behaviors (FTLB), the authors say: “Again, a high number (60%) reported exposure to social media.”  Please clarify that this refers specifically to exposure to tic-related social media content, as mentioned in the original article.

**Response:** Thank you, for this suggestion, we have modified the manuscript accordingly.

 While reading the piece, I wondered if the focus on FTLB (with one section under “Phenomenology and natural history” and a section under “Treatment”) was not excessive, particularly considering that the authors mention that this review article is becoming more voluminous by the year. The studies on the differential diagnosis between tic disorders and FTLB and their comorbidity are in my opinion very fitting and of help from the clinical point of view. However, other articles focused on samples of FTLB alone, as interesting as they are, may be out of scope (maybe with some exceptions).

**Response:** We understand the concern but still in 2023 (and 2024 for that matter), FTLB remain what one could call a “big deal” and, to some extent, a game changer in our field. One current example – that will be incorporated in the 2024 annual highlight paper – is the impact FTLB have and will have on our classification of neurodevelopmental tics, as Dr. Fernández de la Cruz points out (cf. Sarchioto et al., Mov Disord 2024, Jun 18. doi: 10.1002/mds.29868. Epub ahead of print. PMID 38894500.). However, since TS specialists are now also increasingly caring for patients with FTLB on a regular and an extended basis, we believe it is justified to keep articles dedicated solely to FTLB in our yearly review. In a way, even though it is a different entity, FTLB is also an extension of our primary line of work.

 Some acronyms are used but never defined (e.g., ADHD, PUTS, HD). These are most likely well known by the readership but please define for sense of completion the first time that they are mentioned.

**Response: **Thank you for your vigilance, we have modified the manuscript accordingly.

 There are several typos throughout the article. It would be good if the authors could read through and correct them. A few that I caught are listed below:

- Description of Larsh et al, 2023a: please write “higher in adolescent girls than in boys” instead of “then in boys”.
- Larsh et al, 2024: “sex differences in tic severity by retrospectively reviewing Yale Global Tic Severity Scale (YGTSS)”. Something seems to be missing in this clause. Maybe rephrase to “sex differences in tic severity were explored by retrospectively reviewing Yale Global Tic Severity Scale (YGTSS)”?
- Larsh et al, 2024: “but during the pandemic where girls had more severe tics than boys”. Remove the “where”.
- Lewin et al, 2023: “only a subset of questions” instead of “only subset of questions” / “both versions of the scale” instead of “both version of the scale”.
- The acronym FTLB is first used when describing the study by Jack et al. (2023) but it is not defined until the following paragraph/section.
- Nilles et al., 2023: “83 youth” is mentioned twice.
- Colreavy et al, 2023: “sleep patterns were more impacted” instead of “sleep patterns was more impacted”.

**Response:** Thank you for your vigilance, we have modified the manuscript accordingly.

# Reviewer report: Reviewer response for version 1

**DOI:** 10.5256/f1000research.165547.r298065

**Published:** 2024-07-05

**Authors:** Roger L Albin

**Affiliations:** 1 University of Michigan, Ann Arbor, USA

This is a thorough, thoughtful, and easily readable summary of the last year’s contributions to the burgeoning literature on Tourette syndrome and related disorders.  There is a generally nice balance between description and critical assessments. I have only a few minor comments.  The Du et al. study (Annals of Neurology) was based on the concept that there is a substantial developmental deficit of striatal cholinergic interneurons.  There is no *in vivo* evidence for this concept and PET imaging data to the contrary.  In addition, the behavioral methods used in this study have an uncertain relationship to tic behaviors.  The relevance of this work is unclear. I think the Highlights within Highlights section is both accurate and useful. I’d ask the authors to consider a few points related to their “Scrying the future…” section. As a minor point, this section mentions neurosteroids, which I don’t think are mentioned anywhere else.  Why bring this up at the very end?  I’m compelled to respond to the authors’ comments that the typical age of tic onset and male predominance are “mysterious.” I certainly agree these are very relevant phenomena but there is an existing conceptual framework that might be a point of departure for investigations [Refer ref 1].How would organoids contribute to studying “pathophysiology?”  A standard definition of the latter is “the disordered physiological processes associated with disease or injury.” As the authors relate nicely, there is quite a bit of neuroimaging and ephys data on altered brain circuit-network dynamics driving the clinical features of TS. How would a highly reduced preparation like an organoid reproduce these kinds of phenomena?  Indeed, if a behavior is the defining feature of a disorder, how can you know what is relevant in an organoid?

Is the review written in accessible language?

Yes

Are all factual statements correct and adequately supported by citations?

Partly

Are the conclusions drawn appropriate in the context of the current research literature?

Partly

Is the topic of the review discussed comprehensively in the context of the current literature?

Yes

Reviewer Expertise:

Movement Disorders

I confirm that I have read this submission and believe that I have an appropriate level of expertise to confirm that it is of an acceptable scientific standard, however I have significant reservations, as outlined above.

## References

[1] : Tourette Syndrome as a Disorder of the Social Decision Making Network. *Front Psychiatry* .2019;10: 10.3389/fpsyt.2019.00742 742 10.3389/fpsyt.2019.00742 31649568 PMC6792345

# Response

**Published:** 2024-08-05

**Authors:** Andreas Hartmann

**Affiliations:** Pitié-Salpêtrière Hospital, Paris, France

Response: We thank Dr. Albin for his comments. Parenthetically, we would identify this review as an example of the potential benefits of open review: here we have a world expert providing thoughtful and critical feedback that proves equal or superior in utility to those we have received over the years from traditional unsigned reviews. Dr. Albin raises four primary concerns. We summarize them below, each followed by our response.

 The Du et al. study (Annals of Neurology) was based on the concept that there is a substantial developmental deficit of striatal cholinergic interneurons.  There is no *in vivo* evidence for this concept and PET imaging data to the contrary.  In addition, the behavioral methods used in this study have an uncertain relationship to tic behaviors.  The relevance of this work is unclear.

**Response:** The work discussed in the “Animal models” section was motivated by reduced striatal cholinergic interneurons in autopsy samples from five TS patients, a finding not supported by the only *in vivo* human study, a quantitative PET study of 12 young adults with TS and 10 tic-free comparison adults.  We have added this concern to the text.

 I think the Highlights within Highlights section is both accurate and useful. I’d ask the authors to consider a few points related to their “Scrying the future…” section. As a minor point, this section mentions neurosteroids, which I don’t think are mentioned anywhere else.  Why bring this up at the very end? 

**Response:** Articles about neurosteroids in TS were inadvertently omitted from the review section. We have added two sections to the text to correct this omission, one in Pathophysiology / Animal models and one in Treatment / Pharmacological studies.

 I’m compelled to respond to the authors’ comments that the typical age of tic onset and male predominance are “mysterious.” I certainly agree these are very relevant phenomena but there is an existing conceptual framework that might be a point of departure for investigations [Refer ref 1].

**Response:** We were very impressed by Dr. Albin’s unifying theory of TS as a disorder of the brain networks that underlie social decision-making (Albin 2018, 2019). Somehow we missed the important comments there about how that construct can explain the age of tic onset and the high male:female sex ratio. Thank you for directing our attention to these comments ! We have removed those two from the list of interesting mysteries. 

 How would organoids contribute to studying “pathophysiology?”  A standard definition of the latter is “the disordered physiological processes associated with disease or injury.” As the authors relate nicely, there is quite a bit of neuroimaging and ephys data on altered brain circuit-network dynamics driving the clinical features of TS. How would a highly reduced preparation like an organoid reproduce these kinds of phenomena?  Indeed, if a behavior is the defining feature of a disorder, how can you know what is relevant in an organoid?

**Response:** Dr. Albin correctly points out that a blob of brain cells in a dish cannot produce tics, and cannot recapitulate the whole-brain findings from EEG or neuroimaging studies that in any case are more relevant in living humans. Nevertheless, we believe it is appropriate to hypothesize that human brain organoids derived from pluripotent stem cells (hBOs) may at some point reveal useful information relevant to pathophysiology of TS.  Specifically, hBOs can be created from samples of people with and without TS. The two groups of organoids (TS+ / TS−) can be compared in several ways, such as to identify differences in specific neuronal types, neurochemicals or firing patterns. Such differences can direct attention to similar regions of the human brain in autopsy or *in vivo* studies. Novel treatments can be tested to see if they correct any such differences. Specific genes identified as potentially causative in human studies can be examined in hBOs to identify developmental effects caused by manipulating those genes, for instance with optogenetic or CRISPR methods. All of these possibilities are speculative at this moment, and may or may not prove plausible (or ethical, if hBO complexity progresses substantially beyond its current level).  However, similar studies have been conducted successfully in idiopathic autistic disorder (Mariani et al. 2015) and other more dramatic neurodevelopmental conditions (Chiaradia and Lancaster 2020, Wang et al. 2023).  There is no room in the article to discuss this issue adequately, but that seems appropriate for a speculative “what may be in the future” section of a review. We have left the speculation in place in the article text.

**References cited in the response to reviewers**

 Albin RL. Tourette syndrome: a disorder of the social decision-making network. Brain. 2018 Feb 1;141(2):332-347. PubMed PMID: 29053770. Pubmed Central PMCID: PMC5837580. doi 10.1093/brain/awx204.

 Albin RL. Tourette syndrome as a disorder of the social decision making network. Front Psychiatry. 2019;10:742. PubMed PMID: 31649568. Pubmed Central PMCID: PMC6792345. Epub 20191008. doi 10.3389/fpsyt.2019.00742.

 Chiaradia I, Lancaster MA. Brain organoids for the study of human neurobiology at the interface of in vitro and in vivo. Nat Neurosci. 2020 Dec;23(12):1496-1508. PubMed PMID: 33139941. Epub 20201102. doi 10.1038/s41593-020-00730-3.

 Mariani J, Coppola G, Zhang P *, et al.* FOXG1-Dependent Dysregulation of GABA/Glutamate Neuron Differentiation in Autism Spectrum Disorders. Cell. 2015 Jul 16;162(2):375-390. PubMed PMID: 26186191. Pubmed Central PMCID: PMC4519016. doi 10.1016/j.cell.2015.06.034.

 Wang L, Owusu-Hammond C, Sievert D *, et al.* Stem Cell-Based Organoid Models of Neurodevelopmental Disorders. Biol Psychiatry. 2023 Apr 1;93(7):622-631. PubMed PMID: 36759260. Pubmed Central PMCID: PMC10022535. Epub 20230124. doi 10.1016/j.biopsych.2023.01.012.
